# The *Malvastrum Yellow Vein Virus* C4 Protein Promotes Disease Symptom Development and Enhances Virus Accumulation in Plants

**DOI:** 10.3389/fmicb.2019.02425

**Published:** 2019-10-25

**Authors:** Chenchen Jing, Pengbai Li, Jiayuan Zhang, Rui Wang, Gentu Wu, Mingjun Li, Li Xie, Ling Qing

**Affiliations:** ^1^Chongqing Key Laboratory of Plant Disease Biology, College of Plant Protection, Southwest University, Chongqing, China; ^2^Analysis Center of Agrobiology and Environmental Sciences, Zhejiang University, Hangzhou, China

**Keywords:** *Malvastrum yellow vein virus*, C4 protein, symptom determinant, RNA silencing suppressor, viral accumulation

## Abstract

The begomovirus C4 protein is required for disease symptom development during virus infection in host plants. It can reprogram the cell cycle process for more efficient virus accumulation. In this study, we showed that the *Malvastrum yellow vein virus* (MaYVV) C4 protein could cause leaf up-ward curling and flower malformation, and increase virus accumulation in plants using PVX-based transient expression technology. We also demonstrated that, in the presence of its cognate betasatellite DNA (MaYVB), a mutant MaYVV, defective in producing the C4 protein (MaYVVΔC4), caused and alleviated infection in *Nicotiana benthamiana*. Transgenic plants expressing the MaYVV C4 protein showed upward leaf curling and uneven leaf lamina growth. Microscopic analysis showed that the epidermal cells of the C4 transgenic leaves were much smaller than those in the wild type (WT) leaves, and the mesophyll cells size and arrangement of transgenic plants was significantly altered. Inoculation of C4 transgenic plants with MaYVV or MaYVVΔC4 alone or associated with MaYVB showed that the transgenic C4 protein could promote viral and betasatellite accumulation and rescue the accumulation defect of MaYVVΔC4. Other transient expression assays also confirmed that the MaYVV C4 protein could suppress silencing of a GFP gene. In summary, our results indicate that the MaYVV C4 protein is a determinant of disease symptom and viral DNA accumulation. This protein can also function as a suppressor of RNA silencing and alter cell division and expansion.

## Introduction

Geminiviruses are a group of viruses with single-stranded circular DNA genomes that produce twinned virions and belong to the family *Geminiviridae* (Harrison, 1985). Geminiviruses are currently classified into nine genera: *Begomovirus*, *Curtovirus*, *Mastrevirus*, *Topocuvirus*, *Turncurtovirus*, *Eragrovirus*, *Becurtovirus*, *Capulavirus*, and *Grablovirus*, based on their host range, insect vectors and genome organizations (Fauquet et al., 2008; Muhire et al., 2013; Varsani et al., 2014a, b; Brown et al., 2015). Members of the genus *Begomovirus* are either bipartite (i.e., with DNA-A and DNA-B) or monopartite (with a homolog of DNA-A of bipartite begomoviruses). Begomoviruses can infect a wide range of plants and are transmitted by *Bemisia tabaci* (whitefly). Monopartite begomovirus DNA encodes a coat protein (CP) and a V2 protein on its virion-sense strand, and a replication-associated protein (Rep or C1), a replication enhancer protein (Ren or C2), a transcription activator protein (TrAP or C3) and a C4 protein on its complementary-sense strand (Varsani et al., 2014b, 2017).

Virus infection often interferes with plant growth and development by altering host gene expression and various metabolic pathways, leading to the onset of disease symptoms. Virus-encoded proteins are known to interact with specific host factor(s) (Whitham and Wang, 2004; Wang et al., 2018; Wu et al., 2018; Zhou, 2018). Geminivirus *C4* gene is totally nested in *C1* gene in a different reading frame and the corresponding C4 protein has been shown to be a multifunctional protein that can regulate plant cell division, disease symptom development, and virus movement (Deom and Mills-Lujan, 2015; Zeng et al., 2018). Using transgenic plants expressing *Beet curly top virus* (BCTV) C4 protein, Latham and colleagues confirmed that the C4 protein could cause phloem distortion and tumorigenic growth in leaves (Latham et al., 1997). Transgenic *Arabidopsis thalian*a plants expressing the *African cassava mosaic virus* (ACMV) AC4 protein and BCTV C4 protein also showed defects during plant development (Padmanabhan et al., 2005; Mills-Lujan and Deom, 2010). Mutagenesis of the *Cotton leaf curl Kokhran virus* (CLCuKoV) *C4* gene weakened CLCuKoV pathogenicity, alleviated disease symptoms and reduced viral DNA accumulation compared with the wild-type CLCuKoV (Iqbal et al., 2012). A different report showed that *Tomato yellow leaf curl virus* (TYLCV) carrying a mutant *C4* gene was able to replicate in tomato protoplasts and cause systemic infection in *N. benthamiana* plants. However, its accumulation in the infected plants was significantly reduced (Jupin et al., 1994). A recent report indicated that the *Bhendi yellow vein mosaic virus* (BYVMV) C4 protein had no effect on viral DNA replication but could affect virus movement in its host plant (Babu et al., 2018). *Beet severe curly top virus* (BSCTV) mutants carrying new termination codons within the *C4* gene (the nucleotide substitution did not alter the corresponding amino acid sequence of the Rep protein that the *C4* gene overlaps in a different reading frame) accumulated in *A. thaliana* protoplasts and in agro-infiltrated *N. benthamiana* leaves but failed to cause systemic infection in two of the assayed host plants (Teng et al., 2010). Carluccio and others recently reported that the *Mungbean yellow mosaic virus* (MYMV) AC4 protein could bind 21–25 nt siRNAs to counteract virus-induced gene silencing (Carluccio et al., 2018).

The C4 proteins of begomoviruses are also known to affect host cell division by regulating the expression of cell cycle-related genes or by interacting with host receptor-like kinases. For example, BSCTV infection or expression of BSCTV C4 protein in *A. thaliana* caused leaf malformation, upregulated the expression of several cell cycle-related genes: *cycloidea* (*CYC*), *cyclin-dependent kinase* (*CDK*) and *proliferating cell nuclear antigen* (*PCNA*), and downregulated the expression of *CDK inhibitor* and *retinoblastoma-related gene* (*RBR1*) (Park et al., 2010). The Mills-Lujan and Deom publication shows up-regulation of cyclins *cycA1;1* and *cycB1;4*, and cyclin-dependent kinase B2-type namely *CDKB2;2* as early as 24 h after BCTV C4 expression (Mills-Lujan and Deom, 2010). A separate study showed that the expression of BSCTV C4 protein in *A. thaliana* induced the expression of a RING finger protein (RKP) that could affect the stability of the cell cycle inhibitor ICK/KRP, leading to altered plant growth and development (Lai et al., 2010). After phosphorylation and myristoylation, the *Tomato leaf curl Yunnan virus* (TLCYnV) C4 protein was shown to interact with the exportin-α XPO I to facilitate the export of the C4/NbSKη complex from the nucleus to the cell membrane to reduce the nuclear accumulation of NbSKη, to protect NbCycD1;1 from being phosphorylated by NbSKη, presumably to prevent nuclear proteasomal degradation and to induce cell division (Mei et al., 2018a, b).

RNA silencing plays important roles in plant development and plant defense against biotic stresses. RNA silencing can be transcriptional, known as transcriptional gene silencing (TGS), or post-transcriptional, known as post-transcriptional gene silencing (PTGS) (Saijo and Reimer-Michalski, 2013). Successful virus infection in plants depends on an effective viral counter-defense strategy that can defeat host surveillance. To date, many viruses are known to encode one or more proteins that can suppress the host RNA silencing machinery during virus invasion (Burgyan and Havelda, 2011; Szittya and Burgyan, 2013). For example, geminivirus-encoded C4 and AC4 proteins have been shown to suppress TGS or PTGS in plants (Ramesh et al., 2017).

*Malvastrum yellow vein virus* was first isolated from *Malvastrum coromandelianum* in Yunnan Province of China, and also found together with other viruses in field tomato plants showing severe disease symptoms in this province (Zhou et al., 2003; Zhang, 2010). MaYVV infection on *N. benthamiana* induces no visible symptom; when associated with its cognate betasatellite DNA (MaYVB), *N. benthamiana* exhibits downward leaf curling and yellow vein symptoms (Guo et al., 2008). Studies reported in the past decade have significantly advanced our understanding of the functions of geminivirus C4 and AC4 proteins. However, the role of the MaYVV C4 protein during MaYVV infection in plants has not been revealed, so we decided to investigate the functions of MaYVV C4 protein through virus inoculation, transient gene expression and stable transformation. Our results showed that this protein could modulate disease symptom development, viral and betasatellite DNA accumulation and suppress RNA silencing in its host plant. Expression of MaYVV C4 protein in *N. benthamiana* also caused leaf upward curling and malformation phenotype, a reduction in cell size and alter the expression pattern of some cell cycle related genes. We consider that this information is useful for researchers interested in designing breeding strategies for MaYVV-specific resistance in tomato and other solanaceous vegetable crops.

## Materials and Methods

### Plant Materials and Growth Conditions

*N. benthamiana* seed were produced and maintained in the lab. Seeds from 16c transgenic *N. benthamiana* plants were kindly provided by Prof. Hongmei Liu (Shandong Agricultural University, China). Plants were grown inside a growth chamber set at 26°C and a 16 h/8 h (light/dark) photoperiod.

### Plasmid Construction

To generate MaYVV (GenBank No. AJ457824) C4 transgenic plants, the full-length *C4* gene was PCR-amplified from DNA isolated from MaYVV-infected plants using primers C4-F2 and C4-R2 (Supplementary Table S1). The resulting PCR product was cloned into the PCV-N1 binary vector to produce pPCV-N1/C4. To construct the pPVX/C4 vector, the *C4* gene was PCR-amplified using primers C4-F1 and C4-R1. After double digestion with the restriction enzymes *Cla*I and *Sal*I, the PCR product was inserted into the *Cla*I/*Sal*I site of the PVX pGR106 vector, kindly provided by Prof. Jianping Chen (Ningbo University, Zhejiang, China). For gene silencing suppressor activity analysis, the *C4* gene was PCR-amplified using primers C4-F2 and C4-R3 and then inserted into the *Bam*HI/*Pst*I site of the pCHF3 vector to produce pCHF3/C4. To construct a mutant MaYVV plasmid defective in producing the C4 protein, the full-length MaYVV sequence was first PCR-amplified from a MaYVV and MaYVB co-infected plant using primers Y47full-F and Y47full-R. The PCR fragment was cloned into the pGEM-T Easy vector (Promega, Madison, WI, United States) to produce pT-1.0A. After DNA sequencing, a second PCR was performed using the 2 × TransStart FastPfu PCR SuperMix kit (Transgen Biotech, China) and primers Y47C4m-F2 and Y47C4m-R2 to mutate the first two potential translation start codons (ATG) in the *C4* gene to ACG (for threonine) codons. The resulting plasmid was named pT-1.0AΔC4. After double digestion with the restriction enzymes *Eco*RI and *Sal*I, the fragment (referred to as 0.9AΔC4) was inserted into the *Eco*RI/*Sal*I site in the pLH9000 vector to produce pLH9000-0.9AΔC4. Both pT-1.0AΔC4 and pLH9000-0.9AΔC4 were then digested with *Eco*RI enzyme and then ligated together to produce pLH9000-1.9AΔC4 (Supplementary Figure S2). All plasmids were sequenced prior to further use. All the primers used for plasmid constructions are listed in Supplementary Table S1.

### Preparation of C4 Transgenic *N. benthamiana* Plants

Transgenic plants were generated by *Agrobacterium*-mediated transformation as previously described (Small and Leaver, 1988). Seeds from T_0_ transgenic *N. benthamiana* plants were initially grown on MS medium containing 50 μg/mL hygromycin and 100 μg/mL kanamycin (Murashige and Skoog, 1962). The antibiotic-resistant plants were selected and then checked individually for the presence of the *C4* gene by PCR using primers C4-F2 and C4-R2, and by northern blot assays using a *C4* gene specific probe prepared using the DIG High Prime DNA Labeling Kit as instructed by the manufacturer (Roche, Switzerland).

### Agro-Infiltration Assays

The pGR106-based expression vectors were transformed individually into *Agrobacterium tumefaciens* strain GV3101psa. The pCHF3-based expression vectors were transformed individually into *A*. *tumefaciens* strain C58C1, and other expression vectors were transformed individually into *A. tumefaciens* strain GV3101 by electroporation. The transformed bacteria were cultured separately overnight until OD_600_ = 0.8–1.0. The agrobacterium cultures were centrifuged and the pellets were resuspended in induction buffer (10 mM MgCl_2_, 100 mM MES, pH 5.7, and 2 mM acetosyringone in distilled water) followed by 3 h incubation at room temperature. Before infiltration, individual agrobacterium cultures were adjusted to OD_600_ = 1.0. For co-infiltration, the two agrobacterium cultures were individually adjusted to OD_600_ = 2.0 and then mixed at equal volumes. Agrobacterium infiltration was then conducted using plants at the 4–6 leaf stage. All agro-infiltration assays were repeated at least three times, fifteen plants per treatment.

### RT-qPCR

Total RNA was extracted from leaves of agro-infiltrated *N*. *benthamiana* plants using RNAiso Plus reagent as instructed by the manufacturer (TaKaRa, Dalian, China). Complementary DNA (cDNA) synthesis was performed using the PrimeScript^TM^ RT reagent Kit supplemented with a gDNA Eraser (TaKaRa, Dalian, China). Quantitative PCR (qPCR) was then conducted using the NovoStart SYBR qPCR SuperMix Plus kit (Novoprotein, Shanghai, China). Relative gene expression levels were calculated by the 2^–ΔΔCt^ method (Livak and Schmittgen, 2001). Expression of the *NbActin* gene was used as an internal control for the assays. The qPCR reactions were performed using three technical replicates per treatment, and the results shown are the means of three individual experiments.

### Establishment of Standard Curves for the Estimations of MaYVV and MaYVB DNA Copy Numbers

To estimate MaYVV and MaYVB DNA copy number in the infected tissue samples, DNA was extracted from plant tissues using the CTAB method (Cota-Sanchez et al., 2006). The full-length MaYVV or MaYVB sequence was PCR-amplified and inserted into the pGEM-T vector to generate the pMaYVV or pMaYVB plasmid, respectively. A 10-fold serial dilution of the plasmid DNA from 10^9^-10 copies was prepared and used as the standard. qPCR using MaYVV or MaYVB specific primers was designed to produce a 125 bp (MaYVV) or a 126 bp (MaYVB) amplicon, respectively. The optimal reaction mixture was made as follows: 100 ng DNA template, 0.75 μL Y47A-qF (10 μM) and 0.75 μL Y47A-qR (10 μM) primers or 0.50 μL Y47β-qF (10 μM) and 0.50 μL Y47β-qR (10 μM) primers, 10 μL NovoStart SYBR qPCR SuperMix Plus kit (Novoprotein, China) and adjusted to 20 μL of each reaction by RNase-free ddH_2_O. The resulting standard curve for MaYVV appeared linear and had a coefficient of regression *R*^2^ = 0.980 and a calculated slope of −3.181. The resulting standard curve for MaYVB also appeared linear and had a coefficient of regression *R*^2^ = 0.990 and a calculated slope of −3.122. With these two standard curves, the copy numbers of MaYVV or MaYVB in each infected tissue sample could be estimated using the Ct values generated from qPCR assay. The graphs were generated using Origin 9.0 based on the lg (log_10_) value of MaYVV or MaYVB copy numbers in each treatment. The qPCR reactions were performed using three technical replicates per treatment and the results shown are the means from 3 individual experiments, fifteen plants per experiment.

### Southern Blot Assay

Isolated DNA samples (30 μg DNA each) were separated in 1.5% agarose gels by electrophoresis. To visualize the loading of each sample, the agarose gel was stained with GoldView dye (Kehbio, China, Beijing). After transferring the DNA bands to nylon membranes (Roche, Switzerland), the membranes were probed at 42°C using a digoxigenin-dUTP-labeled MaYVV or MaYVB specific probe. Hybridization signals were detected using a Detection Starter Kit II as instructed by the manufacturer (Roche, Switzerland).

### Microscopic Analysis

To view cell nuclei, *N. benthamiana* leaves were cut and stained with a 4′,6-diamidino-2-phenylindole (DAPI) staining solution as instructed by the manufacturer (Biotime, Shanghai, China). The stained leaf tissues were individually mounted in an antifade mounting medium with DAPI on microscope slides and then examined under an inverted fluorescence microscope (ZEISS, Germany). For cell structure and cell size analyses, leaves were cut into small pieces and placed individually in 2.5% glutaraldehyde solution followed by a brief vacuum infiltration. After overnight incubation at 4°C and 3 rinses in a 0.1 M phosphate buffered saline (PBS), the tissues were fixed again in a 1% osmic acid solution for 1–2 h. After 3 rinses in 0.1 M PBS, the tissues were dehydrated in serially diluted ethanol solutions (i.e., 30, 50, 70, 80, 90, and 95%). The dehydrated tissues were embedded individually in epoxy resin. Semi-thin sections (1–2 μm thick) were cut and then stained with a toluidine blue solution prior to examinations under a light microscope (ZEISS, Germany). To observe the size of leaf epidermal cells, small leaf tissues were cut, dried inside a Hitachi HCP-2 chamber (Hitachi, Tokyo, Japan), and surface coated with platinum as instructed by the manufacturer. The tissues were then examined and imaged under a Hitachi SU-8010 scanning electron microscope (SEM) (Hitachi, Tokyo, Japan).

### Western Blot Assay

Total protein was extracted from *N. benthamiana* leaf samples using cell lysis buffer (Biotime, Shanghai, China) and then separated in 15% separating gels through electrophoresis. After transferring the proteins to PVDF membranes, the blots were probed with a GFP-specific polyclonal antibody (CWBIO, Beijing, China) followed by a horseradish peroxidase (HRP)-conjugated goat anti-rabbit IgG secondary antibody (CWBIO, Beijing, China). The detection signal was visualized using the Super ECL plus western blotting kit as instructed by the manufacturer (BIOGROUND, Chongqing, China).

## Results

### Expression of MaYVV C4 in *N. benthamiana* Leaves Caused Upward Leaf Curling and Abnormal Flowering

**FIGURE 1:**
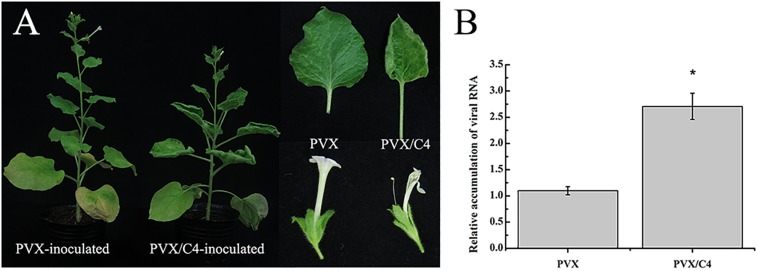
Phenotypes caused by transient expression of the MaYVV C4 protein. **(A)** Phenotypes of *N. benthamiana* plants infected with PVX alone or with PVX/C4. The plants infected with PVX/C4 showed leaf upward curling and abnormal flowering symptoms. Photographs were taken at 30 dpi. **(B)** RT-qPCR analyses of PVX accumulation in the systemic leaves of the PVX- or PVX/C4-infected *N. benthamiana* at 15 dpi. Expression of *Nbactin* was used as an internal control. ^∗^indicates the two treatments differ significantly (*P* value < 0.05 by the Student’s *t*-test). The experiment was repeated three times with similar results.

Geminivirus C4 or AC4 proteins are known as multifunctional proteins modulating virus infection in plants. Because the function of MaYVV C4 protein is unclear, revealing the function of the MaYVV C4 protein would be important to researchers who are interested in various geminiviruses. In this study, we transiently expressed MaYVV C4 protein in *N. benthamiana* plants using a PVX-based vector. At 10 days post inoculation (dpi), the PVX/C4-inoculated plants showed strong mosaic and young leaf distortion symptoms in the leaves and the empty PVX vector-inoculated plants showed mild mosaic symptom in leaves (Supplementary Figure S1). By 30 dpi, upward leaf curling and abnormal flowering phenotypes were observed in the PVX/C4-inoculated plants but not in the PVX-inoculated control plants (Figure 1A). Analysis of PVX accumulation through RT-qPCR showed that more PVX RNA was accumulated in the PVX/C4-inoculated plants compared with that in the PVX-inoculated control plants at 15 dpi (Figure 1B), indicating that the expression of MaYVV C4 protein in plants could affect disease symptom development and virus accumulation.

### Co-infection of *N. benthamiana* Plants With MaYVVΔC4 and MaYVB Caused Milder Disease Symptoms

**FIGURE 2:**
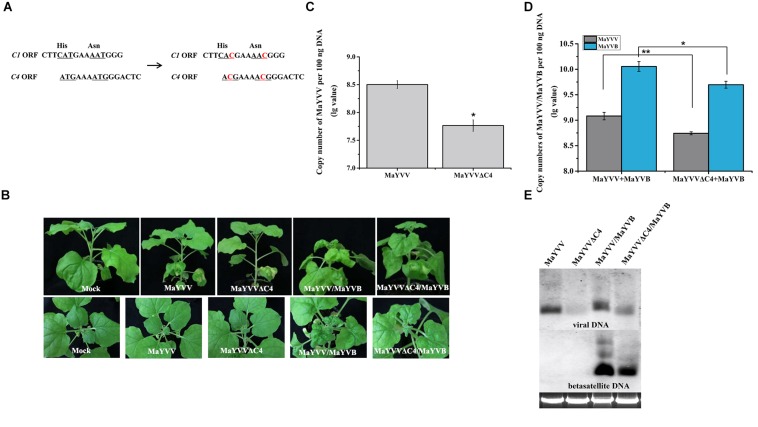
Disease symptoms and viral/betasatellite DNA accumulations in the MaYVV-, MaYVVΔC4-, MaYVV/MaYVB-, and MaYVVΔC4/MaYVB-infected plants. **(A)** A schematic representation of mutations introduced into the *C4* gene. The two potential translation start codons (ATG) in the *C4* gene were mutated into ACG without changing the amino acid sequence of the C1 protein. **(B)** Symptoms induced by inoculations of *N. benthamiana* plants with MaYVV, MaYVVΔC4, MaYVV/MaYVB and MaYVVΔC4/MaYVB at 15 dpi. No obvious symptoms were observed in either MaYVV- or MaYVVΔC4-infected plants. Symptoms in the MaYVVΔC4/MaYVB co-infected plants were delayed and alleviated compared with that shown by the MaYVV/MaYVB co-infected plants. **(C,D)** Quantitative PCR analyses of MaYVV and MaYVB DNA copy numbers in the systemically infected leaves at 15 dpi. ^∗^indicates significantly different between the treatments (*P* value < 0.05 by the Student’s *t*-test); ^∗∗^indicates extremely significant differences between the treatments (*P* < 0.01 by the Student’s *t*-test) by the Student’s *t*-test. **(E)** Southern blot analyses of MaYVV and MaYVB accumulations in the systemically infected leaves at 15 dpi. The blot was probed with specific probes of MaYVV and MaYVB. Genomic DNA was visualized by GoldView staining and was used to show sample loadings.

Because transient expression of MaYVV C4 protein in *N. benthamiana* plants enhanced PVX-induced disease symptoms, we designed MaYVV mutants defective in producing the C4 protein (MaYVVΔC4). Because the C4 protein is encoded by a gene completely embedded in the C1 gene, we used a site-directed mutagenesis strategy to alter the two potential translation start codons (ATG, nucleotide positions 2132–2134 and 2138–2140 in the genome) to ACG (threonine), without changing the C1 protein amino acid sequence (Figure 2A). The stabilities of the two mutated codons were confirmed through PCR amplification using leaf samples collected from the MaYVVΔC4/MaYVB co-infected plants at 35 dpi and DNA sequencing. Because the mutant carrying two mutated codons (MaYVVΔC4-2) was more stable than the mutant carrying only one mutated codon (MaYVVΔC4-1), we used only MaYVVΔC4-2 for this study (Supplementary Figure S3). The mutant construct was then co-inoculated with MaYVB (referred to as MaYVVΔC4/MaYVB hereafter) to *N. benthamiana* plants. The results showed that the MaYVVΔC4/MaYVB co-inoculated plants showed alleviated leaf curling and similar yellow vein symptoms compared with the plants co-inoculated with the MaYVV/MaYVB (Figure 2B). No visible disease symptoms were observed in the plants inoculated with MaYVVΔC4 or MaYVV alone in this study (Figure 2B). qPCR analyses showed that the accumulation of viral DNA and betasatellite DNA in the MaYVVΔC4/MaYVB co-inoculated plants (MaYVV copy number: 10^8.74254 ± 0.02976^, MaYVB copy number: 10^9.69665 ± 0.06764^) were both significantly reduced compared with the MaYVV/MaYVB co-inoculated plants (MaYVV copy number: 10^9.08220 ± 0.07469^, MaYVB copy number: 10^10.05491 ± 0.09639^) at 15 dpi (Figure 2D). Additionally, the accumulation of viral DNA in the MaYVVΔC4-inoculated plants (MaYVV copy number: 10^7.76551 ± 0.10186^) was significantly reduced compared with the MaYVV-inoculated plants (MaYVV copy number: 10^8.50263 ± 0.06892^) (Figure 2C). Similar results were also obtained by southern blot assay using specific probes for MaYVV and MaYVB (Figure 2E).

### Over-Expression of MaYVV C4 Protein in Transgenic *N. benthamiana* Plants Enhanced MaYVV and MaYVB Accumulation

**FIGURE 3:**
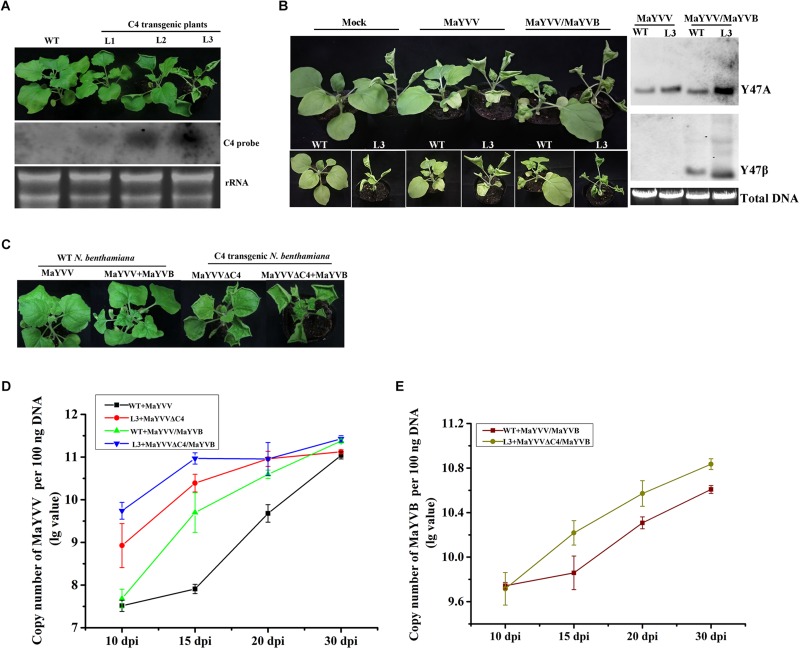
Expression C4 in transgenic plants promoted MaYVV and MaYVB accumulation. **(A)** Phenotypes (upper panel) and northern blot assay (lower panel) of C4 transgenic *N. benthamiana* Line 1, 2, and 3 (L1, L2, and L3). Leaf upward rolling, uneven leaf lamina growth, and plant stunting were observed for the transgenic plants. **(B)** MaYVV and MaYVV/MaYVB infection on the transgenic L3 plants and southern blot assay. Healthy WT and transgenic *N. benthamina* plants were used for comparisons. The plants were photographed at 10 dpi. **(C)** Symptoms caused by MaYVVΔC4 and MaYVVΔC4/MaYVB infection in the transgenic L3 plants. Symptoms caused by MaYVV or MaYVV/MaYVB in the WT *N. benthamina* were used for comparisons. The plants were photographed at 15 dpi. **(D,E)** Detections of MaYVV and MaYVB DNA in systemically infected leaves through qPCR at 10, 15, 20, and 30 dpi, respectively. The experiment was repeated three times with similar results.

To further confirm the function of the C4 protein in MaYVV infection, the MaYVV C4 transgenic plants were generated and confirmed by northern blot (Figure 3A). Transgenic *N. benthamiana* plants expressing MaYVV C4 protein showed upward leaf curling and uneven leaf lamina growth phenotypes similar to the symptoms shown by the plants infected with PVX/C4 (Figure 3A). Inoculation of WT and C4 transgenic *N. benthamiana* line 3 (L3) plants with MaYVV or MaYVV/MaYVB revealed that C4 expression intensifies the MaYVV and MaYVB accumulation in plants at 10 dpi (Figure 3B). Then, we inoculated WT *N. benthamiana* plants with MaYVV or MaYVV/MaYVB and C4 transgenic *N. benthamiana* line 3 (L3) plants with MaYVVΔC4 or MaYVVΔC4/MaYVB to determine whether C4 expression could also rescue accumulation defect of MaYVVΔC4 in plants. The results showed that the MaYVV-inoculated WT *N. benthamiana* plants or the MaYVVΔC4-inoculated L3 transgenic *N. benthamiana* plants did not show visible virus-like symptoms; In contrast, the newly emerged leaves of MaYVV/MaYVB co-inoculated WT *N. benthamiana* plants showed similar leaf curling and yellow vein symptoms with that of MaYVVΔC4/MaYVB co-inoculated L3 transgenic plants (Figure 3C). Quantification PCR analysis using samples harvested at 10, 15, 20, and 30 dpi showed that the accumulation of viral DNA in the MaYVVΔC4-inoculated L3 transgenic *N. benthamiana* plants was significantly higher than that in the MaYVV-inoculated WT *N. benthamiana* plants (Figure 3D). When the L3 transgenic plants were co-inoculated with MaYVVΔC4/MaYVB, the accumulation of both viral and betasatellite DNA were significantly increased compared with that in the WT *N. benthamiana* plants co-inoculated with MaYVV/MaYVB (Figures 3D,E). All these results indicated that C4 expression could facilitate virus and betasatellite accumulation and strongly rescue the accumulation defect of MaYVVΔC4.

### Expression of MaYVV C4 in *N. benthamiana* Altered Leaf Cell Division and Expansion

**FIGURE 4:**
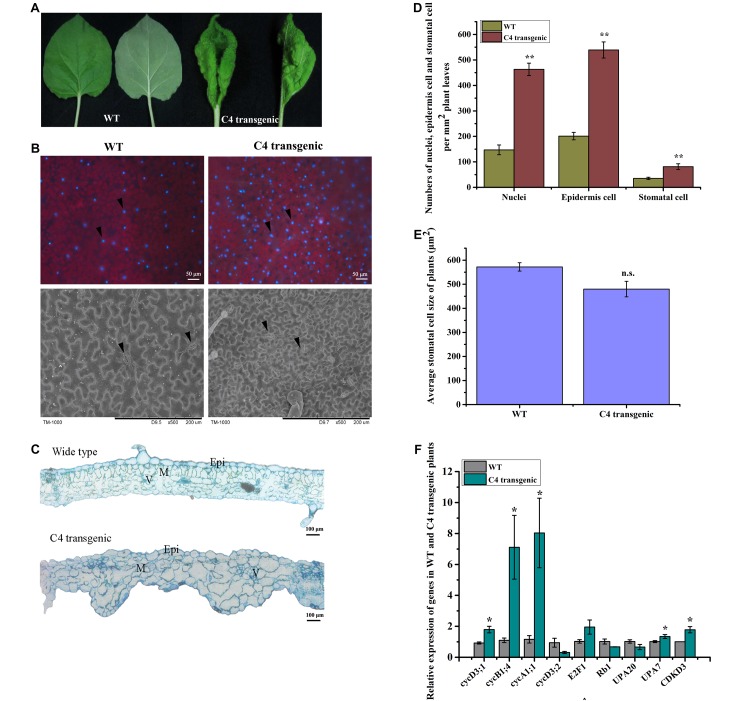
Morphological studies of the WT and C4 transgenic plant leaves. **(A)** Images of leaves showing the adaxial or abaxial side of a WT (left) and a C4 transgenic *N. benthamiana* leaf (right). **(B)** Nuclei of leaf cells were stained with DAPI. The stained leaves were examined and photographed under a fluorescent inverted microscope (upper panel). Black arrowheads indicate the DAPI-stained nuclei. The abaxial side of a WT and a C4 transgenic leaf were examined and photographed under a scanning electron microscope. Black arrowheads indicate stomatal cells. **(C)** Cross sections were cut from an embedded WT or a C4 transgenic leaf tissue and then examined under a light microscope. Epi, epidermal cells; M, mesophyll cells; V, vasculature. **(D)** Statistical analyses of nuclei, epidermis cell and stomatal cell numbers per mm^2^ of WT and C4 transgenic plant leaves, the data was averaged from 4 samples of each treatment. ^∗∗^indicates extremely significant difference between the treatments (*P* value < 0.01 by the Student’s *t*-test). **(E)** Statistical analysis of stomatal cell size of WT and C4 transgenic plant leaves. n.s., indicates no significance between the treatments (*P* value > 0.05 by the Student’s *t*-test). **(F)** RT-qPCR analysis of cell division and expansion-related genes in WT and C4 transgenic plants at 4–6 leaf stage. ^∗^indicates significantly different between the treatments (*P* value < 0.05 by the Student’s *t*-test).

In this study, leaves of the C4 transgenic *N. benthamiana* plants showed upward curling and uneven leaf lamina growth (Figure 4A). To determine whether over-expression of C4 in *N. benthamiana* could also alter cell division and expansion, we examined leaf surfaces, and leaf cross sections by microscopy. DAPI staining results showed that the average number of stained nuclei (approximately 149 nuclei per mm^2^) in the WT *N. benthamiana* leaves was much less than that (approximately 462 nuclei per mm^2^) in the C4 transgenic leaves (Figures 4B,D). Additionally, the average size of C4 transgenic leaf epidermal cells was much smaller than that in the WT leaves. To further confirm this finding, we examined the WT or C4 transgenic leaves under a scanning electron microscope. The results showed that the average number of epidermal cells in WT *N. benthamiana* leaves was approximately 201 per mm^2^ (*n* = 4) and approximately 539 per mm^2^ (*n* = 4) in the C4 transgenic leaves. The average number of stomatal cells in the WT *N. benthamiana* leaves was approximately 35 per mm^2^ (*n* = 4) and approximately 81 per mm^2^ (*n* = 4) in the C4 transgenic leaves (Figures 4B,D). The average size of stomatal cells in the WT (approximately 572 μm^2^) and C4 transgenic (approximately 480 μm^2^) leaves shares no significance (Figure 4E). Examination of resin-embedded leaf cross sections showed that the size and the arrangement of mesophyll cells in C4 transgenic leaves were significantly altered compared with that in the WT leaf cross sections, such as uneven leaf lamina growth, disordered and larger palisade and spongy cells, and some crowds of mesophyll cells (Figure 4C). Analysis of the expression of several genes involve in cell division and expansion: *cycD3;1*, *cycB1;4*, *cycA1;1*, *cycA3;2, CDKD3, E2F1, Rb1, upa20* and *upa7*, showed that the expression of the *C4* gene in plants significantly upregulated the expression of *cycD3;1*, *cycB1;4*, *cycA1;1*, *upa7* and *CDKD3*. No significant changes were found in *cycD3;2*, *E2F1*, *Rb1* and *upa20* expression between the WT and the C4 transgenic plants (Figure 4F).

### MaYVV C4 Protein Could Suppress RNA Silencing in Plants

**FIGURE 5:**
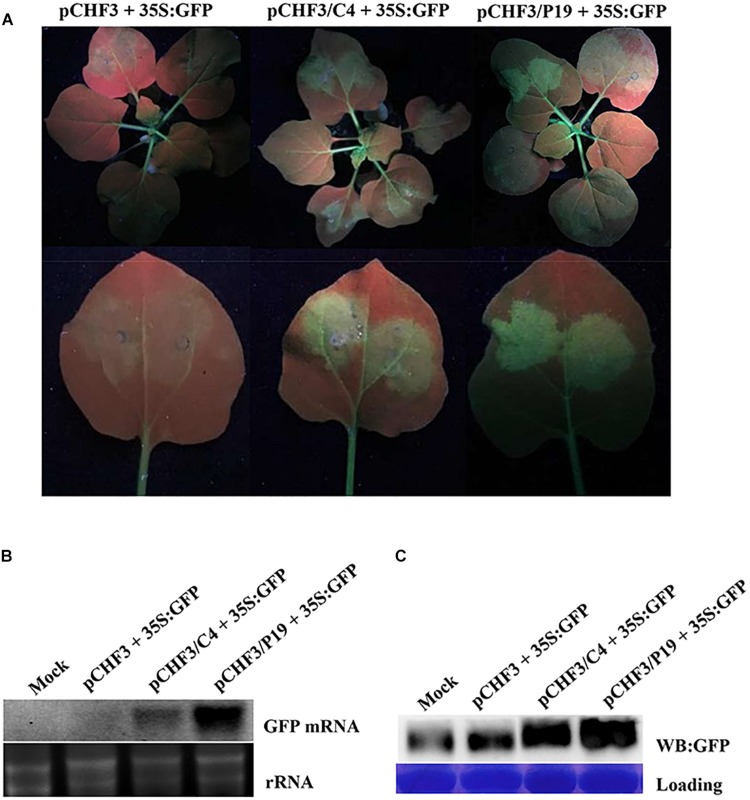
MaYVV C4 protein can suppress PTGS. **(A)** 16c *N. benthamiana* leaves were agro-infiltrated with pCHF3 and pCHF3/eGFP (pCHF3 + 35S: GFP), pCHF3/C4 and pCHF3/eGFP (pCHF3/C4 + 35S: GFP) or pCHF3/P19 and pCHF3/eGFP (pCHF3/P19 + 35S: GFP). The infiltrated leaves were photographed at 3 dpi. One infiltrated leaf of each plant was detached and photographed again (lower panel). **(B)** Northern blot analysis of *GFP* mRNA accumulation in different agro-infiltrated leaves. GoldView-stained rRNA is used to show sample loadings. **(C)** Western blot analysis of GFP protein accumulation in different agro-infiltrated leaves using a GFP specific antibody followed by a HRP-conjugated goat-anti-rabbit antibody. The Coomassie brilliant blue-stained rubisco was used to show sample loadings.

Post-transcriptional gene silencing is one of the key players in the plant anti-viral defense system. To counteract host anti-virus defenses, viruses have also evolved to encode specific suppressors of RNA silencing (Li and Wang, 2019). To investigate whether the MaYVV C4 protein could also function as an RNA silencing suppressor, *Agrobacterium* cultures, carrying pCHF3/eGFP (referred as 35S: GFP), pCHF3/C4, pCHF3/p19 [a vector expressing the *Tomato bushy stunt virus P19* gene (Qiu et al., 2002; Qu and Morris, 2002)] or pCHF3, were agro-infiltrated into the leaves of 16c transgenic *N. benthamiana* plants. At 3 dpi, both treatments co-infiltrated with pCHF3/C4 + 35S: GFP and pCHF3/p19 + 35S: GFP showed strong green fluorescence in the infiltrated areas (Figure 5A). In contrast, the leaves of the 16c transgenic plants co-infiltrated with pCHF3 + 35S: GFP (control plants) showed no obvious green fluorescence in the infiltrated areas. Northern blot and western blot assay results confirmed the presence of *GFP* mRNA and GFP protein in both treatments infiltrated with pCHF3/C4 + 35S: GFP and pCHF3/p19 + 35S: GFP (Figures 5B,C). No visible amount of *GFP* mRNA and little GFP protein were detected in the pCHF3 + 35S: GFP co-infiltrated leaves.

## Discussion

Geminiviruses infect a wide range of food crops and weeds worldwide. Geminivirus-infected plants often show strong disease symptoms, including leaf upward curling, leaf yellowing, enation and plant stunting (Cui et al., 2004; Li et al., 2005, 2018). Among them, some monopartite begomoviruses often found associating with betasatellites in infected field samples (Zhou, 2013). C4 and AC4 proteins of several geminiviruses have been identified as disease symptom determinants (Fondong, 2019). In this study, we determined that the MaYVV-encoded C4 protein is also involved in disease symptom development. The MaYVV C4 protein can regulate plant cell division and expansion and viral and betasatellite DNA accumulation, and it can suppress RNA silencing in its host plant.

Leaves are important plant organs and function primarily in photosynthesis. Leaves expand in three directions: adaxial–abaxial, proximal–distal, and meddle–lateral axes. Over-expression of MaYVV C4 protein in *N. benthamiana* leaves using a PVX-based vector or through stable transformation resulted in leaf upward curling and enation, mainly due to the uneven leaf lamina growth. Co-infection with MaYVVΔC4/MaYVB of *N. benthamiana* leaves resulted in alleviated leaf curling, indicating that the C4 protein is responsible for the strong disease symptoms observed in the field. Previous studies have demonstrated that the introduction of mutations into the gene encoding the begomovirus C4 protein could reduce virus accumulation in plants (Ismayil et al., 2018). In this study, we found that this C4 protein facilitates virus and betasatellite accumulation. This conclusion is further supported by the result showing that the C4 transgenic plants inoculated with MaYVV, MaYVVΔC4 alone or together with its cognate betasatellite DNA accumulated more viral and betasatellite DNA than that in the WT *N. benthamiana* plants inoculated with MaYVV alone or with MaYVV/MaYVB.

RNA silencing is a sequence homology-dependent RNA degradation mechanism that occurs in the cytoplasm (Sijen and Kooter, 2000; Hammond et al., 2001). Plant viruses are inducers of RNA silencing and are also targets of RNA silencing. Numerous plant viruses are known to encode RNA silencing suppressors, including BCTV V2 protein, RSV NS3 protein and TLCYnV C4 protein (Xie et al., 2013; Kim et al., 2017; Luna et al., 2017). In this study, after agro-infiltration of 16c plants with pCHF3/C4 + 35S: GFP, green fluorescence remained in the leaves by 3 dpi. In contrast, the 16c plants agro-infiltrated with pCHF3 + 35S: GFP lost GFP signal, suggesting they were silenced. Consequently, we conclude that the MaYVV C4 protein is also a suppressor of PTGS. However, whether MaYVV C4 protein functions in TGS remains to be uncovered. *Cotton leaf curl Multan virus* (CLCuMuV)-encoded C4 protein suppresses both TGS and PTGS by inhibiting S-adenosyl methionine synthetase (SAMS) activity to enhance virus infection in plants, and silencing of *NbSAMS2* reduces both TGS and PTGS (Ismayil et al., 2018). MaYVV C4 protein shares 49.02% identity with CLCuMuV C4 protein, and in previous transcriptome analysis of MaYVV-*C4* transgenic plants, 3 transcripts that predicted to be *SAMS* were significantly down-regulated when compared with wild type (WT) *N. benthamiana* plants (data not shown), but the relationship between C4-induced *SAMS* down-regulation and the PTGS suppressor function remains to be uncovered. TYLCV C4 protein inhibits the intercellular spread of RNAi by interacting with the intracellular domain of BAM1 and BAM2 at the plasma membrane and plasmodesmata, the cytoplasmic connections between plant cells (Rosas-Diaz et al., 2018). MaYVV C4 protein shares high identity (66.67%) with TYLCV C4 protein, whether MaYVV C4 functions as a suppressor in a similar manner like TYLCV C4 remains testifying.

Because viruses hijack host factors to complete their life cycles in plants, they often cause disruptions of cell division and cell cycle patterns. For example, BSCTV C4 induces RING finger E3 ligase RKP expression, which has been suggested to regulate the plant cell cycle (Lai et al., 2010). TLCYnV C4 protein was reported to interact with NbSKη and increase the nuclear-accumulation level of NbCycD1;1 to induce cell divisions (Park et al., 2010; Mei et al., 2018a, b). To date, how the MaYVV C4 protein regulates cell cycles remains unclear. In this study, the expression of MaYVV C4 reduced epidermal cell size, altered mesophyll cell arrangement, and upregulated the expression of different cyclin genes and *CDKD3*. Different cyclins are known to function differently during cell cycle transitions. Upon BCTV C4 protein expression, cyclins *cycA1;1* and *cycB1;4*, and cyclin-dependent kinase B2-type, *CDKB2;2*were up-regulated (Mills-Lujan and Deom, 2010). The three genes would be considered mitotic markers for their up-regulation in the G2/M transition and the M phases of the cell cycle (Dewitte and Murray, 2003). MaYVV C4 protein significantly induced the expression of *cycA1;1* and *cycB1;4* and presumably contribute to disordered cell cycle and abnormal growth of MaYVV-C4 transgenic plants. Over-expression of *cycD3;1* in plants was reported to induce smaller pavement cells than in WT plants (Walter et al., 2003). Therefore, we speculate that the MaYVV C4 protein-induced expression of *cycD3;1* may play an essential role in the transgenic plants phenotype formation. D-type cyclins are the primary mediators of the G1/S transition. Active CDKA-CYCD complexes are known to phosphorylate and inactivate the Rb protein to prevent its association with E2F, which is important for blocking the activation of E2F-regulated genes (Scofield et al., 2014). The E2F-regulated genes include those involved in the S phase and other growth and cell-cycle processes (Scofield et al., 2014). The MaYVV C4 protein did not alter the expression of *E2F1* and *Rb1*, it is possible that *E2F1* and *Rb1* may not be involved in symptom development of MaYVV C4. The effector protein of *Xanthomonas* spp., namely, AvrBs3, was shown to induce the expression of *upa20*, a transcription factor and a master regulator of cell size (Kay et al., 2007). It was reported that *upa20* could activate the expression of *upa7*, which encodes a putative α-expansin (Kay et al., 2007). In this study, we found that expression of the MaYVV C4 protein upregulated the expression of *upa7* but not *upa20*. Thus the reduced cell size of C4 transgenic plants is not associated with the expression of *upa20* and the involvement of *upa7* needs further study for confirmation.

Collectively, the results presented in this paper indicated that the MaYVV C4 protein regulates viral and betasatellite DNA accumulation in plants, acts as an RNA silencing suppressor, and can alter host cell division and expansion. Further investigations on how the MaYVV C4 protein interacts with host factor(s) will benefit researchers who are interested in breeding MaYVV-specific resistance in crops and the management of this virus in the field.

## Data Availability Statement

The datasets generated for this study can be found in GenBank, AJ457824.

## Author Contributions

LQ and CJ conceived and designed the experiments. LQ, CJ, PL, RW, ML, GW, LX, and JZ performed the experiments. CJ and LQ wrote the manuscript.

## Conflict of Interest

The authors declare that the research was conducted in the absence of any commercial or financial relationships that could be construed as a potential conflict of interest.
