# Supplementary figures and images for: The Malvastrum Yellow Vein Virus C4 Protein Promotes Disease Symptom Development and Enhances Virus Accumulation in Plants
Source: Front Microbiol. 2019 Oct 25;10:2425. doi: 10.3389/fmicb.2019.02425 (PMC6823909; doi:10.3389/fmicb.2019.02425)

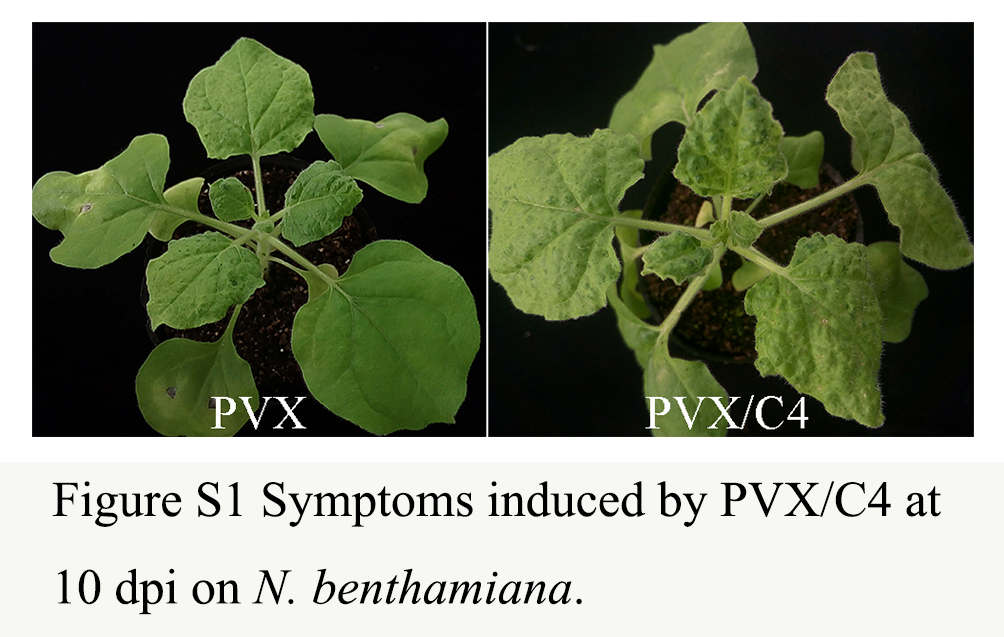

Supplement: Supplementary file 1 [file Image_1.TIF]

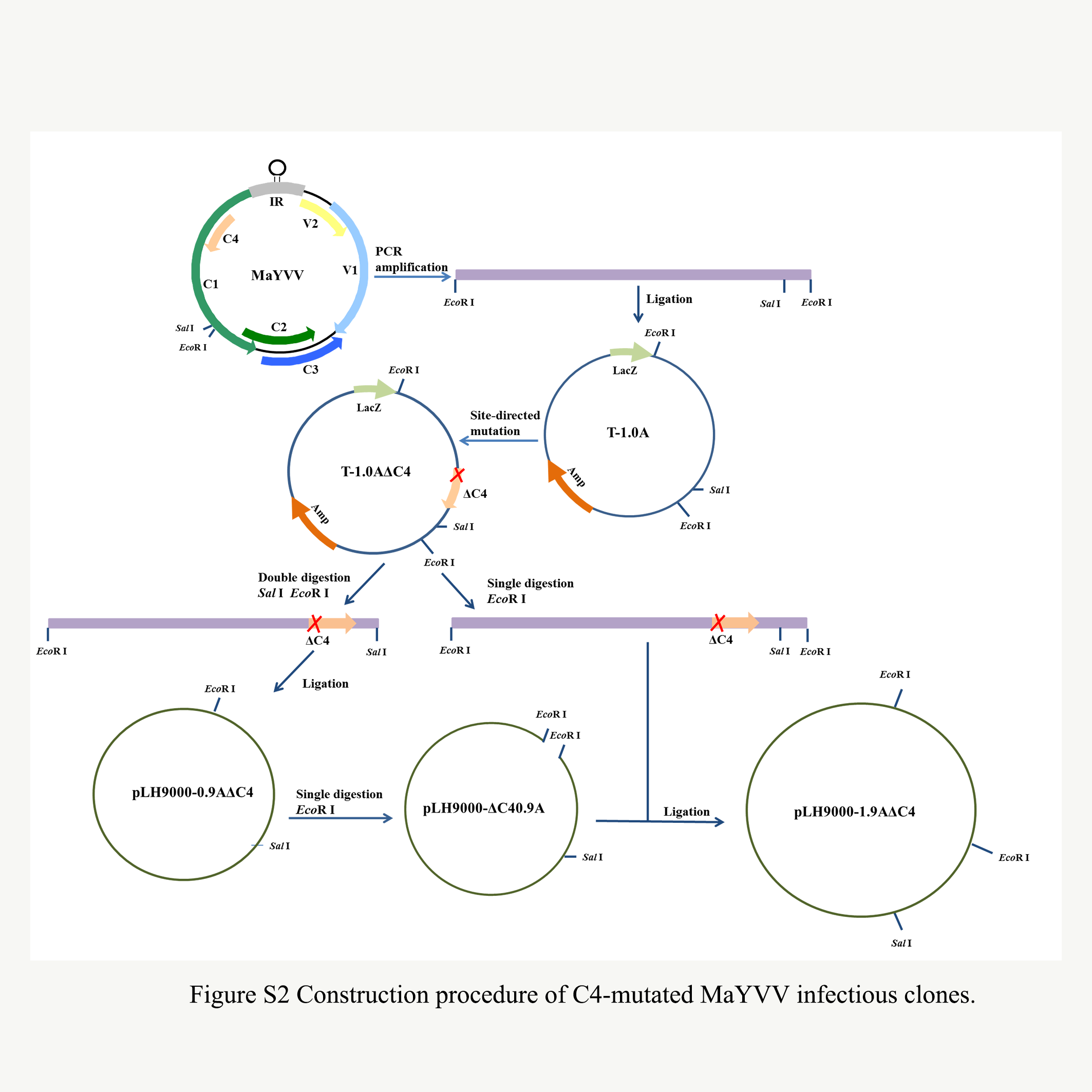

Supplement: Supplementary file 2 [file Image_2.TIF]

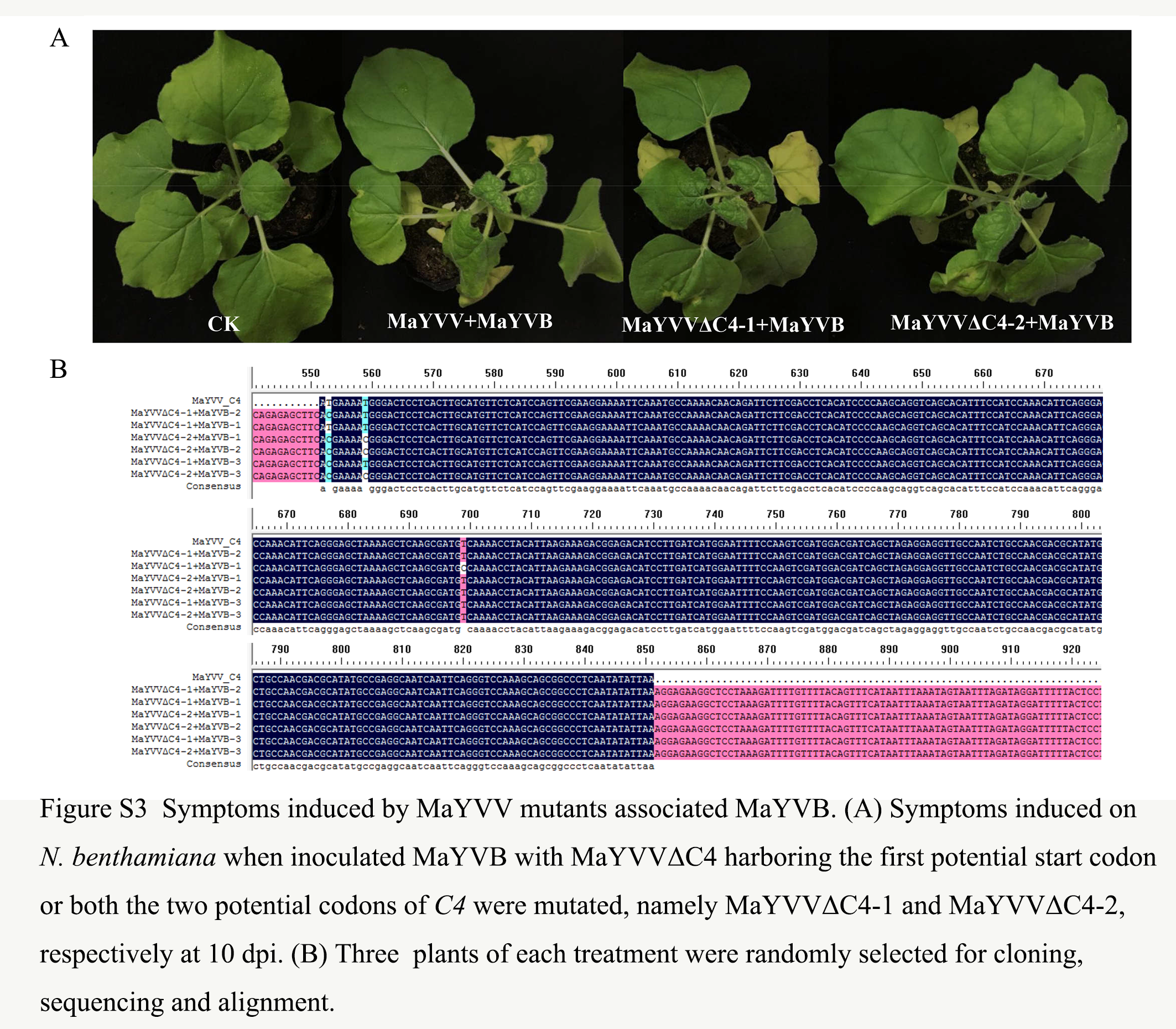

Supplement: Supplementary file 3 [file Image_3.TIF]
