# Supplementary material for: The Malvastrum Yellow Vein Virus C4 Protein Promotes Disease Symptom Development and Enhances Virus Accumulation in Plants
Source: Front Microbiol. 2019 Oct 25;10:2425. doi: 10.3389/fmicb.2019.02425 (PMC6823909; doi:10.3389/fmicb.2019.02425)
Supplement: Supplementary file 4 [file Table_1.DOCX]

Table S1 Sequence of primers used in this study

| **Primer name** | **Sequence(5’-3’)** |
| --- | --- |
| C4-F1 | AATCGATATGAAAATGGGACTCCTC |
| C4-R1 | GGTCGACTTAATATATTGAGGGCC |
| PVXcp-F | CTCAACTACCACGAAAACTGC |
| PVXcp-R | TGTGCACACCTCTTTGATTGC |
| Nb-actin-F | CTTGAAACAGCAAAGACCAGC |
| Nb-actin-R | CATCCTATCAGCAATGCCCG |
| Y47C4m-F1 | GAGGAGTCCCATTTTCgTGAAGCTCTCTGC |
| Y47C4m-R1 | cGAAAATGGGACTCCTCACTTGCATGTTCT |
| Y47C4m-F2 | GAGGAGTCCCgTTTTCgTGAAGCTCTCTGC |
| Y47C4m-R2 | cGAAAAcGGGACTCCTCACTTGCATGTTCT |
| Y47A-qF | CATGATCAAGGATGTCTCCGTC |
| Y47A-qR | ACAACAGATTCTTTGACCTCAC |
| Y47β-qF | CCGTTGTAGGCGAATAAGAAATTG |
| Y47β-qR | CAGGACACCAGCGATCATCAAG |
| Y47ASB-F | CGTCGTTGCTGTAGACCTTTG |
| Y47ASB-R | CAATTATGCCATTTAGGTCCTGA |
| Y47BSB-F | ACTGACTGCCTTGACTGGATTTG |
| Y47BSB-R | CGTACACACCCCATGTATTAGG |
| C4-F2 | GGATCCATGAAAATGGGACTC |
| C4-R2 | GAGCTCTTAATATATTGAGGGC |
| eGFP-F | GACCACATGAAGCAGCACGAC |
| eGFP-R | CCGTCCTCCTTGAAGTCGATG |
| Y47AC4mdet-F | GCCCACGCTTCAAGTTCTTC |
| Y47AC4mdet-R | CTTACTAAAGAAGAGGCACTTTC |
| Y47full-R | GAATTCCTCGACGAGAAAGA |
| Y47full-F | GAATTCTTTATAGCTGCTATTG |
| C4-R3 | CTGCAGTTAATATATTGAGGGCC |
